# The Medical Diploma Craze

**Published:** 1885-07

**Authors:** 


### THE MEDICAL DIPLOMA CRAZE.

In the June issue of the *American Veterinary Review* is a paper by our colleague, Prof. Liautard, upon this subject, which is worthy of attention. It would seem as if many of the younger men in the profession had gone mad in their desire to hide their regular calling under the, to them, more resonant title of "medical doctor."

The title one has is simply the announcement to the public that one is, or should be, capable of offering it the services of an educated professional. The more we complicate this title by adding on a whole alphabet of letters, which are entirely meaningless to the public, the more we detract from the dignified position that we should assume.

How many Americans know what F.R.C.V.S., or what is still worse, H.F.R.C.V.S. means?

We are beginning to demand that the law take some recognition of our profession, but if our legislators are to face this babel of titles they will certainly be a long time before they tell who is a Veterinarian, and who not.

The M.D. attached to a Veterinarian's name is neither worth the money, or time or study it takes to obtain it. The regular medical profession looks upon such a person as an interloper, who is ever willing to step outside his profession to make a dollar and the public think him an M.D., who, not being successful in practice, becomes a "horse doctor," because he was not fit for anything else.

It required no such addition to his name to enable Bouley to become President of the Society in France, a position in the highest degree honorable. Gerlach, Leisering, Roell, Toussaint, Chauvaeux, Dick, Percivall, and others have needed no M.D. to lift them into the heaven of immortals. Let our work tell what we are, and if in the course of time it is of such value to the world that some noted university gives us the honorary title of M.D., as has been the case in Europe, then we have indeed earned it, but the M.D. of an American medical school is of far less value to us Veterinarians than our own honestly won diplomas, because we must first ask how it was got and from what school it was obtained, before we are sure it is a sign of any increased educational ability.

If Veterinarians desire to complete their education in the very things they most lack, as we stated in our last editorial let them attend some of the post graduate schools, where they can get all they need—but no humbug-giving diplomas—in a more practical form and at less expense than at any medical school. Let us be honest and stand on our own feet and not try to fly with borrowed wings.

There should be a convention of delegates from all these schools, and one title—Doctor of Veterinary Medicine—adopted by all. B.

